# New Monomer Based on Eugenol Methacrylate, Synthesis, Polymerization and Copolymerization with Methyl Methacrylate–Characterization and Thermal Properties

**DOI:** 10.3390/polym12010160

**Published:** 2020-01-08

**Authors:** Abdel-Basit Al-Odayni, Waseem Sharaf Saeed, Ahmed Yacine Badjah Hadj Ahmed, Ali Alrahlah, Abdullah Al-Kahtani, Taieb Aouak

**Affiliations:** 1 Chemistry Department, College of Science, King Saud University, P. O. Box 2455, Riyadh 11451, Saudi Arabia; aalodayni@ksu.edu.sa (A.-B.A.-O.); wsaeed@ksu.edu.sa (W.S.S.); ybadjah@ksu.edu.sa (A.Y.B.H.A.); akahtani@ksu.edu.sa (A.A.-K.); 2 Engineer Abdullah Bugshan Research Chair for Dental and Oral Rehabilitation, College of Dentistry, King Saud University, Riyadh 11545, Saudi Arabia; 3 Restorative Dental Sciences Department, College of Dentistry, King Saud University, Riyadh 11545, Saudi Arabia

**Keywords:** eugenol methacrylic ester, poly(eugenol methacrylate), poly(eugenol methacrylate-co-methyl methacrylate), synthesis, characterization, thermal properties, direct analysis in real time (DART)

## Abstract

Poly(eugenyl-2-hydroxypropyl methacrylate) (PEUGMA), poly(methyl methacrylate) (PMMA) and poly(eugenyl-2-hydroxypropyl methacrylate-co-methyl methacrylate) (PEUGMA-co-MMA) were synthesized by a free radical polymerization route in the presence of azobisisobutyronitrile. EUGMA was synthesized by etherification of the eugenol phenolic hydroxyl group with glycidyl methacrylate. Polymers and copolymers were characterized using size exclusion chromatography, Fourier transform infrared, and nuclear magnetic resonance. The effects of the encumbering substituent on the thermal behavior of the polymers and copolymers were studied by differential scanning calorimetry, thermogravimetry (TG) and direct analysis, using real-time, time-of-flight mass spectroscopy (DART-ToF-MS) methods. The results obtained revealed that for PEUGMA, the average molecular weight was 1.08 × 10^5^, and increased slowly with the decrease in the EUGMA content in the copolymer. The order of the distribution of dyads comonomer units in the copolymer chains estimated by the Igarashi method based on the reactivity ratio does reveal a random distribution with a tendency toward alternation. The glass transition temperature of PEUGMA (46 °C) increased with the MMA content in the copolymer, and those of the copolymer fit well with the Johnston’s linearized expression. The TG analysis of pure PEUGMA revealed a significantly high thermal stability compared to that of PMMA. During its degradation, the preliminary decomposition was at 340 °C, and decreased as the MMA units increased in the copolymer. The DART-ToF-MS analysis revealed that the isothermal decomposition of PEUGMA led to a regeneration of raw materials such as EUGMA, GMA and EUG, in which the maximum amount was achieved at 450 °C.

## 1. Introduction

Methacrylate polymers containing epoxyl groups have attracted several researchers in different domains. This is because these materials are endowed with the well-balanced properties of epoxy and methacrylate, and the epoxy functional groups can improve the performance of the resulting materials through crosslinking reactions. Based on this principle, poly(glycidyl methacrylate) (PGMA) is utilized the most in this family, and has been the subject of a large number of applications; these are used in surface modification [1,2,3,4,5,6], adhesives [7,8], compatibilizers for polymer blends [9,10,11,12], electrolytes [13] and biochemistry [14,15,16]. In addition, PGMA has been used as the base material for the design of various compounds via copolymerization or grafting [17,18,19,20,21,22,23,24].

Poly(alkyl methacrylate)s are amorphous, transparent, and can be easily converted into numerous semifinished products such as films, rods, tubes and sheets. The decent mechanical properties, thermal properties, high transparency and excellent weather ability, render poly(methyl methacrylate) to be the most widely used in practical applications. The physical and chemical properties of poly(methyl methacrylate)s are characterized by different factors such as the molecular weight and strength of intermolecular forces, as well as the regularity of the polymer structure, microstructure and flexibility. In poly(alkyl methacrylate)s, as the size of the alkyl group increases, the distance between the polymer molecules increases, and intermolecular attraction is reduced. Thus, as the side chain length increases, the softening point decreases; the polymers become elastic at progressively lower temperatures. However, when the number of carbon atoms in the side chain exceeds 12, side chain crystallization becomes significant, and the polymers become less elastic. Indeed, Czech et al. [25] reported that chain branching enhances hardness, while chain elongation increases plasticization.

The nature of the alkyl group in poly(alkyl methacrylate)s also determines the mode of the thermal decomposition of the polymer. For example, when poly(methyl methacrylate) is heated above 200 °C, an unzipping reaction results in an almost quantitative production of monomer. However, according to Grassie [26], polymers of higher esters tend to degrade only partially to monomer, and lead to poorer yields of the monomer. According to Czech et al. [27], the general mechanism of the thermal degradation of poly(alkyl methacrylates) consists of random main-chain scission reactions. The principal products resulting from pyrolysis occurring between 200 and 400 °C are methacrylate monomers.

The thermal degradation behavior of PGMA was studied by Zulfiqar et al. [28] using thermogravimetry in dynamic nitrogen and thermal volatilization analysis (TVA) under vacuum. The resulting product was predominantly the monomer, indicating that a depolymerization reaction occurred during the process. Minor products arising from ester decomposition such as acrolein, allyl alcohol, glycidol, carbon dioxane, isobutene and propene were also revealed.

During our literature survey, we found that there were no investigations of the polymerization and copolymerization of this monomer with other acrylic monomers. Given the importance of the material that can be generated by the polymerization and copolymerization of this monomer with other acrylics, a detailed study on the subject, particularly with regard to the synthesis and physicochemical properties of the products, deserves to be conducted.

Our contribution in this work is to synthesize a new bulky monomer such as eugenyl-2-hydroxypropyl methacrylate (EUGMA) via an etherification route through eugenol phenolic hydroxyl group and glycidyl methacrylate (GMA) using the epoxy ring opening method. The obtained monomer was then homopolymerized and copolymerized with MMA in different compositions via a radical polymerization technique. The influence of the bulky substituent on the molecular weight and microstructure of the obtained polymers and copolymers were investigated. To reach this goal, different parameters were studied, such as the reactivity ratios and the order of distribution of dyads comonomer units in the copolymer chains were undertaken, using nuclear magnetic resonance (NMR) and UV-visible. The thermal behavior of polymers and the influence of the bulky units in the copolymers were also in detail investigated by differential scanning calorimetry (DSC) and thermogravimetric analysis (TGA). 

A direct analysis in real time (DART) was also employed for the first time to study the isothermal decomposition of the synthesized polymers and copolymers.

## 2. Experimental

### 2.1. Materials

Eugenol (EUG) (purity 98.5%), glycidyl methacrylate (GMA) (purity 98%), triphenylphosphine (TPP) (purity 99%), hydroquinone (HQ) (purity ≥ 99%), 1, 4-dioxan (purity 99%), ethyl acetate (purity ≥ 99.5%), hexane (purity 95%), tetrahydrofuran (THF) (purity 98%), methyl methacrylate (MMA), and Azobisisobutyronitrile (AIBN) (purity 98%) were purchased from Sigma-Aldrich (Taufkirchen, Germany). Monomers were freed from hydroquinone (inhibitor) by distillation under reduced pressure. AIBN was further purified several times by recrystallization in methanol. All liquid chemicals were stored under nitrogen gas before use.

### 2.2. Analysis

The molecular weight of the prepared polymers and copolymers was measured in THF at 30 °C by size exclusion chromatography (SEC). SEC involves using a Varian apparatus equipped with a 880-PU high-performance liquid chromatography (HPLC) pump refractive index, ultraviolet (UV) detectors and TSK gel columns (JASCO, Easton, MD, USA). Fourier-transform infrared spectra were recorded on a Nicolet iS10 spectrometer (Thermo Scientific, Madison, WI, USA) equipped with an attenuated total reflection (ATR; diamond crystal) accessory. The spectra were obtained over a region of 4000–500 cm^−1^ at room temperature and acquired with a total of 32 scans per spectrum and resolution of 2 cm^−1^. Proton nuclear magnetic resonance (^1^HNMR) and carbon-13 nuclear magnetic resonance (^13^CNMR) spectra were respectively taken at 400 and 200 MHz on a spectrometer (Delta-NMR, Jeol Resonance, Tokyo, Japan) using CDCl_3_ as a solvent. UV-visible analysis was carried out using a Hitachi U-2910 spectrometer. The comonomer composition in each copolymer was estimated by this method using a calibration curve plotted from the absorbance measurements of poly(eugenyl-2-hydroxypropyl methacrylate) (PEUGMA)/poly(methyl methacrylate) (PMMA) blends in chloroform as a solvent.

Differential scanning calorimetry (DSC) thermograms were obtained by a Shimadsu DSC 60 (Shimadzu, Kyoto, Japan) system previously calibrated with indium. Then, 8–10 mg of the tested homopolymers and copolymers was packed in aluminum DSC pans before being placed in a DSC cell and heated under nitrogen gas from 30 to 200 °C at a heating rate of 20 °C·min^−1^. TGA measurements were carried out on a Shimadsu TGA 60 under dynamic nitrogen gas. Afterward, 4–10 mg of polymer or copolymers was carefully loaded into the TGA aluminum pan and heated from 25 to 600 °C at a heating rate of 20 °C·min^−1^.

The isothermal decomposition of polymers and copolymers was detected using an Accu-ToF LC-plus JMS-T100 LP mass spectrometer (JEOL, Tokyo, Japan) equipped with a direct-analysis-in-real-time (DART) ion source (IonSense, Saugus, MA, USA), without any prior preparation of the sample. The volatile components of the extract were evaporated in a stream of helium heated between 200 and 450 °C, then ionized by the excited metastable helium atoms, before entering the ion source of the time-of-flight mass spectrometer. In the positive ionization mode, each molecule was transformed into either a protonated ion [M + H]^+^ or a nonprotonated radical molecular ion [M^+^]. Since the ionization process is considered “soft,” little or no fragmentation occurs, such that each peak in the spectrum corresponds to a given compound. The experimental conditions used for analyzing the samples by direct analysis, using real-time, time-of-flight mass spectroscopy (DART-ToF-MS) were: vacuum analysis 1.3 × 10^−5^ Pa, heating and ionization gas, ring lens voltage 4 V, peaks voltage 500 V and mass resolution range between 3600 and 4900.

### 2.3. Synthesis

#### 2.3.1. Eugenyl-2-Hydroxypropyl Methacrylate (EUGMA)

**Scheme 1:**
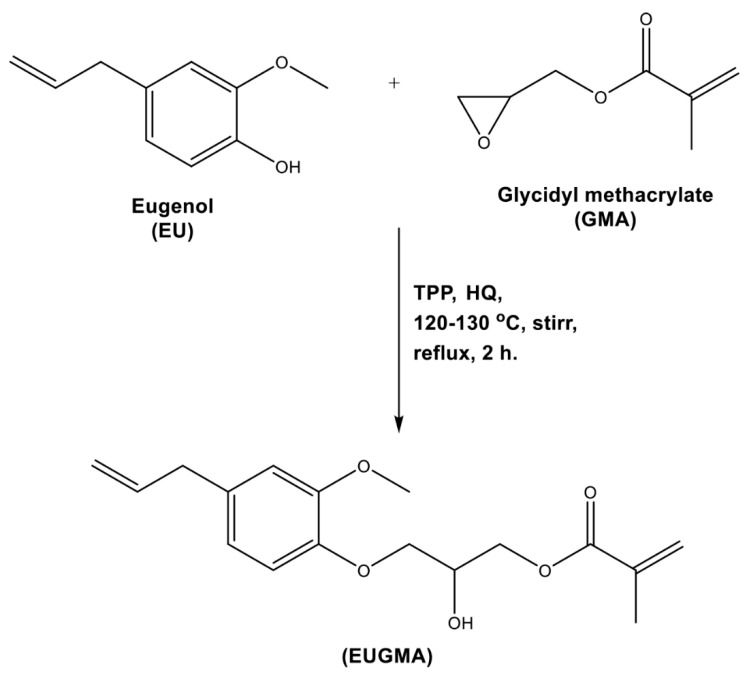
Synthesis of the eugenyl-2-hydroxypropyl methacrylate (EUGMA) monomer.

The targeted eugenol-based methacrylate monomer, eugenyl-2-hydroxypropyl methacrylate (PEUGMA), was synthesized via etherification of the eugenol phenolic hydroxyl group with a GMA epoxy-ring opening route. The reaction was carried out under a nitrogen gas atmosphere. The reactivity of the vinyl and allyl groups of GMA and EUG was inhibited by hydroquinone (HQ), while the etherification was catalyzed by triphenylphosphine (TPP), according to the reaction in Scheme 1.

EUG and GMA in equal mole ratios were placed in a three-necked round-bottom flask equipped with a magnetic stirrer, reflux condenser and nitrogen inlet. HQ at 0.5 wt % and TPP at 0.1 wt % from the total mixture were then added under stirring to this reaction mixture and heated at 120 °C for about 2 h. The reaction progression was monitored through thin layer chromatography (TLC) using an ethyl acetate/hexane (7:3) system as a mobile phase. The analyte spots were visualized using a UV light in short-wave mode. After the reaction was completed, the mixture was left to cool to room temperature. The obtained product was purified from residual reactants, TPP and HQ using flash column chromatography with silica gel (60 mesh) and an elution system of ethyl acetate/hexane (7:3). Based on the TLC analysis, the fractions containing pure EUGMA were collected, and then the solvent was completely removed using a rotary evaporator. The obtained EUGMA was dried at 30 °C under vacuum until constant weight. Finally, a light-yellow liquid of 69 wt % was yielded from this reaction, and the structure of the obtained monomer was confirmed by FTIR and ^1^H and ^13^C NMR analysis. Pure EUGMA isolated was then kept at 8 °C before use.

#### 2.3.2. Synthesis of Polymers and Copolymers

**Scheme 2:**
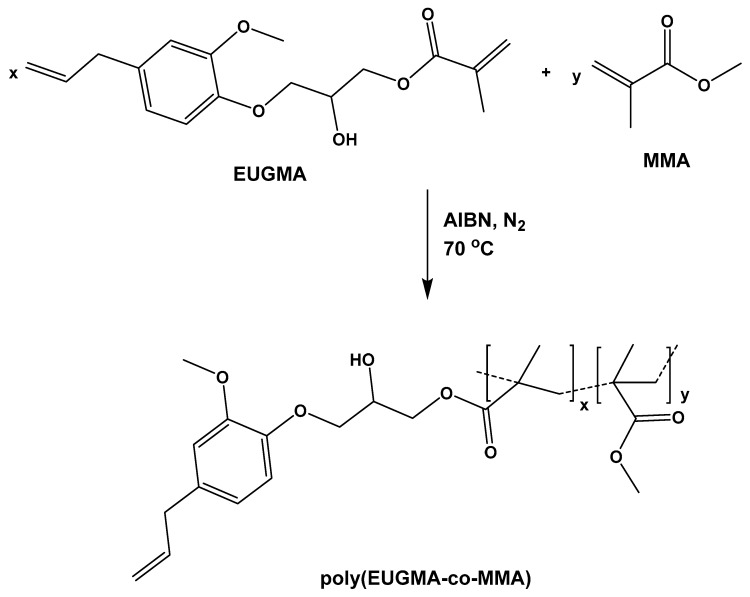
Synthesis of poly(eugenyl-2-hydroxypropyl methacrylate-co-methyl methacrylate) (PEUGMA-co-MMA) copolymers.

PEUGMA, PMMA homopolymers, and PEUGMA-co-MMA copolymers were synthesized under a nitrogen gas atmosphere via the free radical polymerization route in 1,4-dioxan using azobisisobutyronitrile (AIBN) as initiator at 70 °C. The copolymerization reaction of EUGMA with MMA is shown in Scheme 2.

The very viscous solution obtained was poured into an excess *n*-hexane from which the precipitated copolymer was filtered and purified two times by repeating the precipitation in *n*-hexane. It is important to note that no gel or microgel was obtained after the microfiltration of each polymeric solution, indicating the absence of allylic–allylic and/or allylic–vinylic intermolecular reactions leading to the production of crosslinking homopolymer and copolymer. The resulting polymer was dried in a vacuum oven at room temperature for 48 h. In order to determine the reactivity ratio, the conversion rate was as limited as possible to values less than or equal to 12% relative to the amount of the starting monomer by limiting the time of the polymerization reaction at 20 min. Under these conditions, a series of copolymers containing different MMA content ranging between 25 and 75 mol % was synthesized by the same method. The preparation conditions are summarized in Table 1.

## 3. Results and Discussion

### 3.1. Synthesis and Characterization of EUGMA

**Figure 1:**
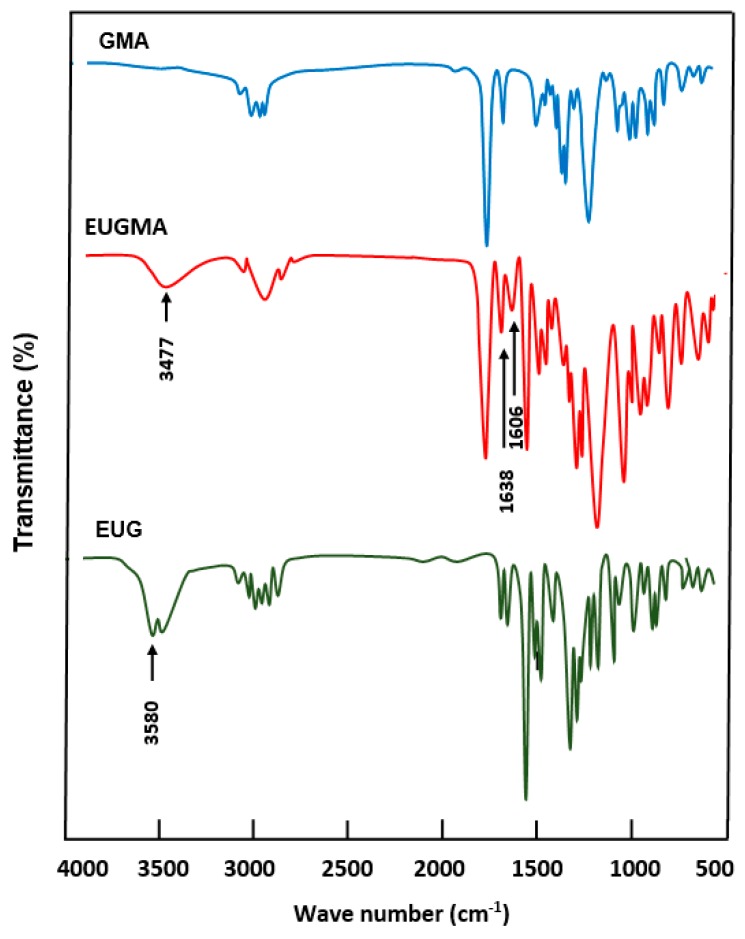
Fourier-transform infrared spectroscopy (FTIR) spectra of GMA, EUG and EUGMA.

**Figure 2:**
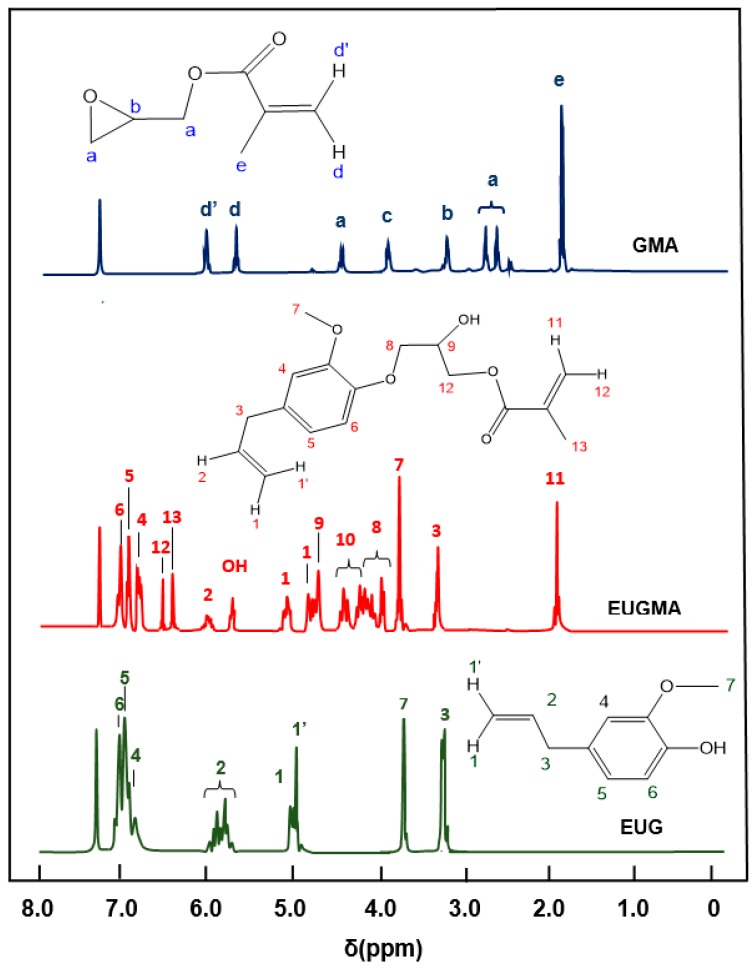
Proton nuclear magnetic resonance (^1^H NMR) spectra of EUG, GMA, and EUGMA in CDCl_3_.

**Figure 3:**
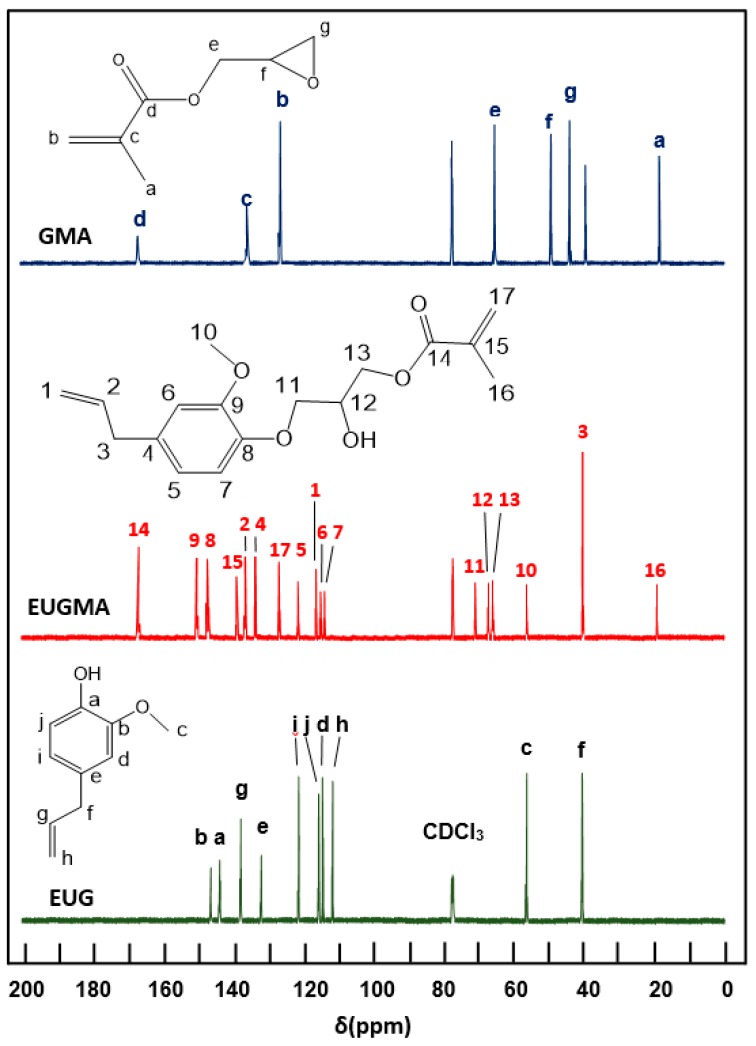
Carbon-13 nuclear magnetic resonance (^13^C NMR) spectra of EUG, GMA, and EUGMA in CDCl_3_.

The FTIR spectrum of EUGMA and those of their components are shown in Figure 1. Indeed, the EUGMA spectrum reveals the total disappearance of the absorption band centered at 3580 cm^−1^, corresponding to the phenolic hydroxyl group of Eugenol and the appearance of a new absorption band at 3477 cm^−1^ attributed to the secondary hydroxyl group characterizing the synthesized monomer. On the other hand, the absorption band which is localized at 1606 cm^−1^ is assigned to the C=C of the allylic group, and that at 1638 cm^−1^ is attributed to the C=C vinylic group, thus indicating the conservation of the radical polymerization site. The ^1^HNMR analysis confirmed the previous results obtained by FTIR analysis by comparing the EUGMA spectrum with those of their components, as shown in Figure 2. Indeed, the structure of this monomer was confirmed by the disappearance of the absorption peak at 9.9 ppm attributed to the proton (14) of the hydroxyl phenolic group and the appearance of the signal at 5.77 ppm attributed to the proton of the secondary hydroxyl group. Practically no change in the other signals attributed to the two components was observed in the EUGMA spectrum, indicating the conservation of the polymerization site as noted in the previous FTIR analysis. The ^13^CNMR analysis also confirmed the EUGMA structure through a comparison of this prepared monomer spectrum with those of the starting reagents, as shown in Figure 3. As in the case of the preceding analyses, the appearance of the peak centered at 65.0 ppm was assigned to the carbon (12) carrying the hydroxyl group. The simultaneous disappearance of the peak at 144.2 ppm, which was attributed to the carbon (a) carrying the hydroxyl linked to the aromatic group, was an indication of the formation of monomer in addition to other signals present in the reactants that remained unchanged.

### 3.2. Synthesis of Homopolymers and Copolymers

The results of the homopolymerization of EUGMA and MMA and their copolymerization, are given in Table 2. As can be seen from these data, the copolymerization yield achieved under the same conditions, the total amount of monomer did not exceed 12 wt %.

#### 3.2.1. SEC Analysis

**Figure 4:**
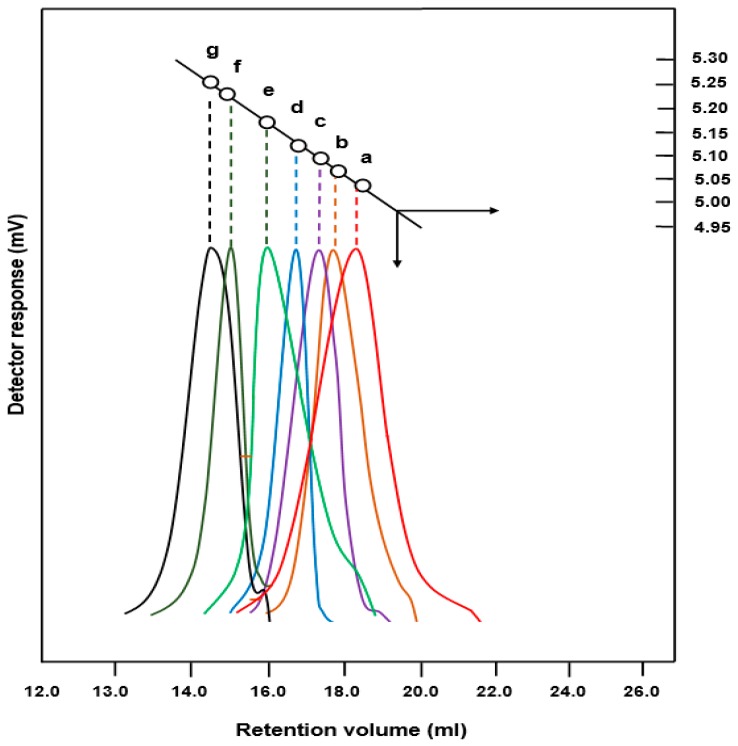
Size exclusion chromatography (SEC) chromatograms: (a) PEUGMA; (b) PMMA; (c) PEUGMA-*co*-MMA25; (d) PEUGMA-*co*-MMA35; (e) PEUGMA-*co*-MMA50; (f) PEUGMA-*co*-MMA65 and (g) PEUGMA-*co*-MMA75.

**Figure 5:**
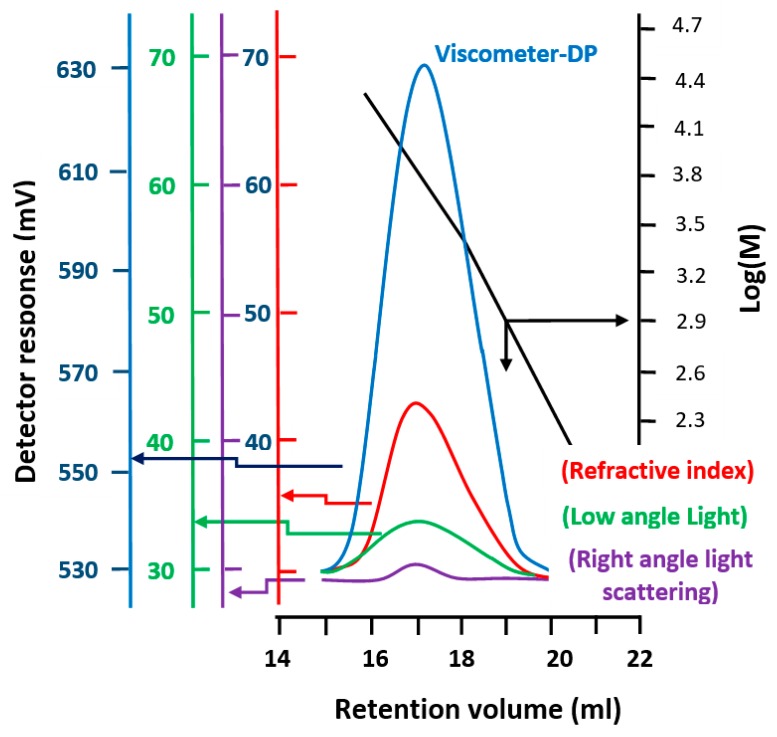
Data treated from the SEC chromatogram of PEUGMA as example of synthesized polymers.

The profiles of the SEC chromatograms of the PEUGMA, PMMA, and PEUGMA-co-MMA with different MMA content are gathered in Figure 4. An example of the molar masses deducted from the data treated is presented in Figure 5.

The molar masses and the polydispersity index of the polymers and copolymers determined by SEC analysis are grouped in Table 2. As can be seen from these data, the molecular masses were relatively high and decreased as the starting EUGMA increased in the reaction mixture. This phenomenon can be attributed to the steric hindrance of the monomer substituent. Indeed, such a substitute group covers the active site and consequently limits the access of the monomer to the macroradical. This phenomenon also decreases the kinetics of polymerization. Indeed, a rapid increase in the viscosity of the medium was caused by the incorporation of heavy monomeric units in the growing chains. The values of the polydispersity index (I), which vary randomly, are relatively high in characterizing the radical polymerization reactions, which are mainly owing to the transfer reactions.

#### 3.2.2. Structure

##### FTIR Analysis

**Figure 6:**
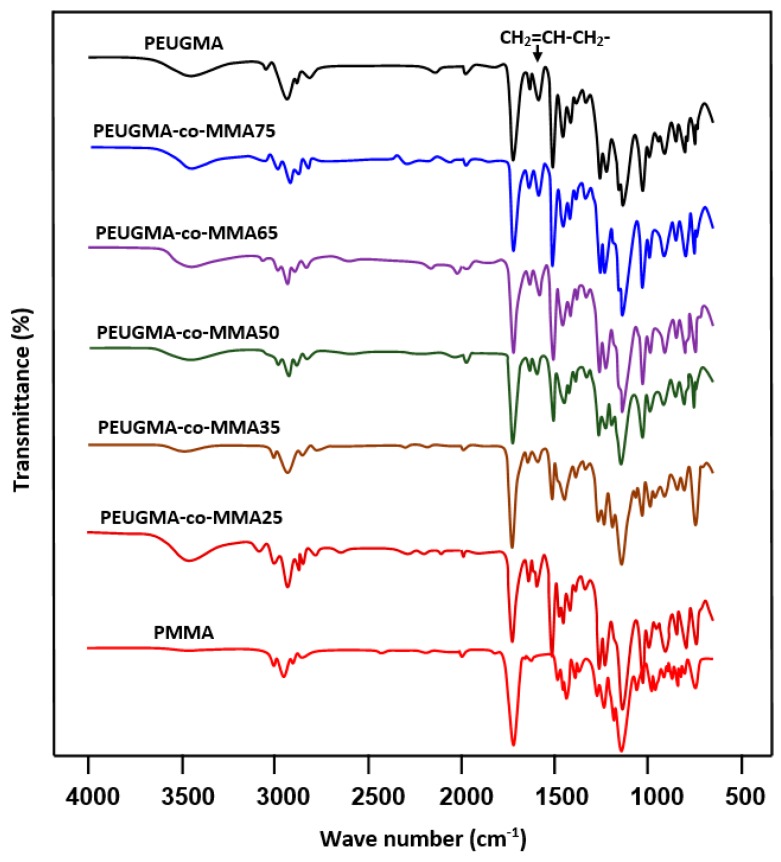
FTIR spectra of PEUGMA-co-MMA copolymers and those of their corresponding homopolymers MMA and EUGMA.

A comparison of the FTIR spectra of the synthesized copolymer with those of the two corresponding homopolymers is shown in Figure 6. All PEUGMA-co-MMA spectra show the same bands common to both comonomers, indicating their real presence in the copolymer. The absence of new peaks and the lack of disappearance of the absorption peaks at 1618 cm^−1^, which is assigned to the isolated olefinic bonds indirectly linked to the phenyl group, indicate that the structure of the copolymer is not affected during the copolymerization process. As noted in the Section 2.3.2, no gel or microgel resulting from possible crosslinking reaction occurred between the allylic–allylic double bonds or allylic–vinylic double bonds were observed. 

This seems predictable, because the allyl radicals are much more stable than the corresponding vinyl radicals, and that propagation is quickly terminated by hydrogen abstraction from an allylic monomer. According to different authors [29,30,31], the self-termination by allylic hydrogen atom abstraction is often referred to as allylic degradative chain transfer. The allylic monomer radical stabilizes itself by resonance which explains the slow polymerization of allylic monomers and the relatively low molecular weight of the polymers.

Similar difficulties were observed when copolymerizing allylic monomers with vinylic monomers. Consequently, to polymerize allylic monomers, large quantities of free radical initiators are required, resulting in relatively large amounts of terminal initiator end groups and residual initiator decomposition products. For this reason, no poly(eugenol) synthesized by free radical polymerization was reported in the literature. On the contrary, according to Fujisawa and al. [32], eugenol has been found as a retarder against polymerization MMA using benzoyl peroxide or AIBN as our initiator.

##### NMR Analysis

**Figure 7:**
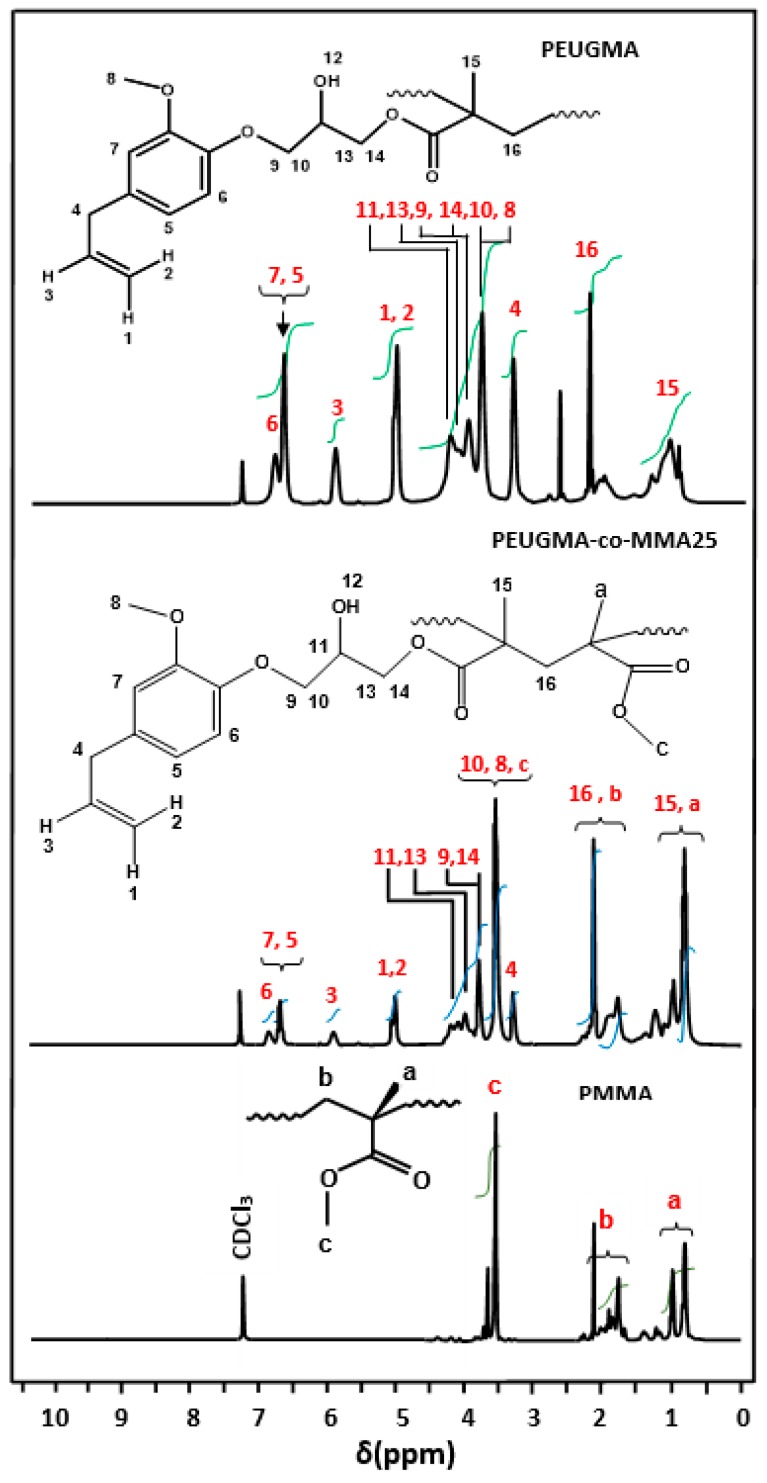
Comparative ^1^HNMR spectra of PMMA, PEUGMA and PEUGMA-co-MMA25.

The structure of homopolymers and copolymers were highlighted by ^1^H and ^13^C NMR analysis through the comparison of their spectra with those of their monomer components in which the PEUGMA-co-MMA25 is presented as an example. The ^1^HNMR spectrum of this copolymer in Figure 7 reveals a total disappearance of the signals at 4.8, 5.0 and 5.9 assigned to the three vinylic protons (1, 1′ and 2) observed in the EUGMA spectrum (Figure 2) and a co-presence of all other signals of the monomers spectra including those of the allylic C=C.

The comparison of the signal area of the two vinylic protons (1,2) with that of the two protons of carbon-4 and before the polymerization process, whose ratio is close to one, remains unchanged, indicating that the allylic double bond is not affected during the reaction.

This observation confirms the stability character of the C=C bond allyl group during the free radical polymerization as described by the mechanism proposed by Bartlett [29,30] and Vilodina [32]. Basing on the areas of the signals between 0.60 and 1.30 ppm, which are attributed to the three protons –CH_3_ (15,a) common to both monomeric units and those at 3.33 ppm, assigned to the distinct protons attached to the methylenic carbon-4, the determination of the comonomer units in each copolymer was possible using Equation (1)

(1) $$\mathrm{EUGMA}\left(mol\%\right)=\frac{6\delta_{CH_{2}\left(4\right)}}{2\delta_{CH_{3}\left(15,a\right)}}\times 100\;\;\;\;\;\;\;\;\;\;$$

where *δ_CH_*_2_(4) and *δ_CH_*_3_(15,*a*) are the areas of the two protons of carbon-4 and that grouped with the carbon-15 and carbon-a, respectively, and the estimated results are grouped in Table 3.

**Figure 8:**
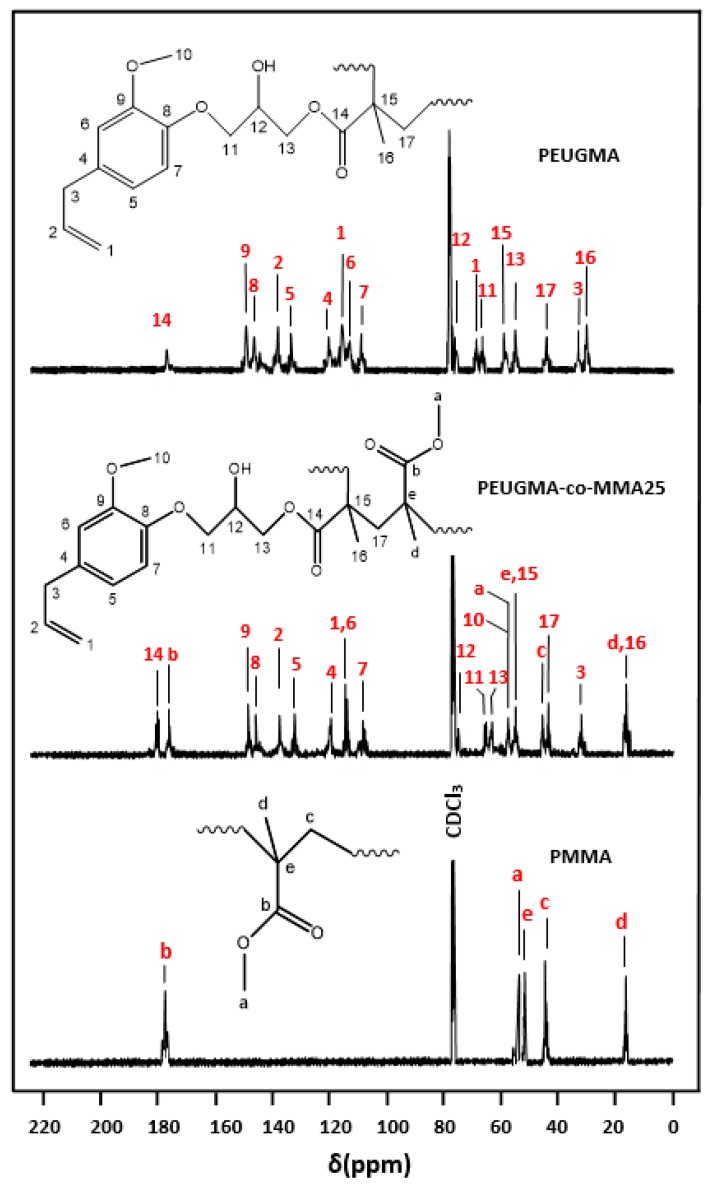
Comparative ^13^CNMR spectra of PMMA, PEUGMA and PEUGMA-co-MMA25.

Comparable results were also obtained by the ^13^CNMR analysis in Figure 8. Indeed, the structure of this copolymer is confirmed by the disappearance of the characteristic signals at 140 ppm (carbon-15) and 128 ppm (carbon-17) of the vinylic group of the EUGMA monomer observed (Figure 3), which are the site of the polymerization. On the other hand, all the other carbons are well present on this spectrum including those of the allylic double bonds of the EUGMA unit.

##### UV Analysis

The PEUGMA-co-MMA composition was confirmed by UV-visible analysis through the maximum absorption band of the PEUGMA phenyl group at 277 nm using the calibration curve indicating the variation of the absorbance versus the PEUGMA/PMMA composition. The results obtained are gathered for comparison in Table 3. As can be seen from these data these values confirm those obtained by the ^1^HNMR method, and their arithmetic averages were used to estimate the reactivity ratio.

##### Comonomer Distribution

**Figure 9:**
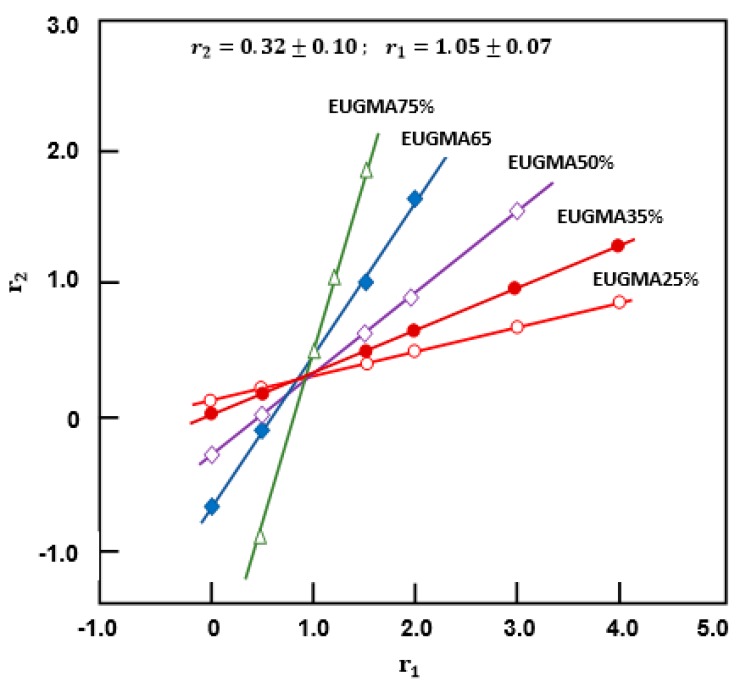
Curves indicating the variation of r_2_ vs. r_1_ obtained from Mayo–Lewis equation.

The distribution of EUGMA and MMA units in the PEUGMA-co-MMA chains was estimated from the reactivity ratios (*r*_1_ and *r*_2_) for the EUGMA/MMA copolymerization process. The values of these parameters were calculated from the comonomer feed ratios and the copolymer compositions obtained previously by nuclear magnetic resonance (NMR) (Table 3) through the Mayo–Lewis equation [33]. Using this method, *r*_2_ values were plotted as a function of the various assumed values of *r*_1_ according to Equation (2) for each experiment with different feed and copolymer compositions. The subscripts (1) and (2) indicate EUGMMA and MMA monomers.

(2) $$r_{2}=\frac{f_{1}}{f_{2}}[\frac{F_{2}}{F_{1}}(1+\frac{f_{1}}{f_{2}}r_{1})-1]$$

where *f_i_* is the mole fraction of the monomer (*i*) in the feed, and *F_i_* is the mole fraction of the monomer unit (*i*) in the copolymer. The reactivity ratios are estimated from the intersection of the straight lines of the curves indicating the variation of *r*_2_ vs. *r*_1_ of Figure 9 traced from Equation (2). The area in which most lines intersect can be given as an experimental error range.

As can be seen from these curve profiles, the variations of *r*_2_ vs. *r*_1_ resulted in linear curves, and their intersections reveal *r*_2_ and *r*_1_ values of 0.32 ± 0.10 and 1.05 ± 0.07, respectively. Using the same data of Table 3, the reactivity ratios were also determined for comparison by the traces plotted using the Fineman–Ross (3) [34] and Kelen–Tüdős (4) [35] equations.

(3) $$G=Hr_{1}-r_{2}$$

where

(4) $$G=\frac{f_{1}(2F_{1}-1)}{(1-f_{1})F_{1}}\;\mathrm{and}\;H=\frac{f_{1}^{2}(1-F_{1})}{{\left(1-f_{1}\right)}^{2}F_{1}}$$

**Figure 10:**
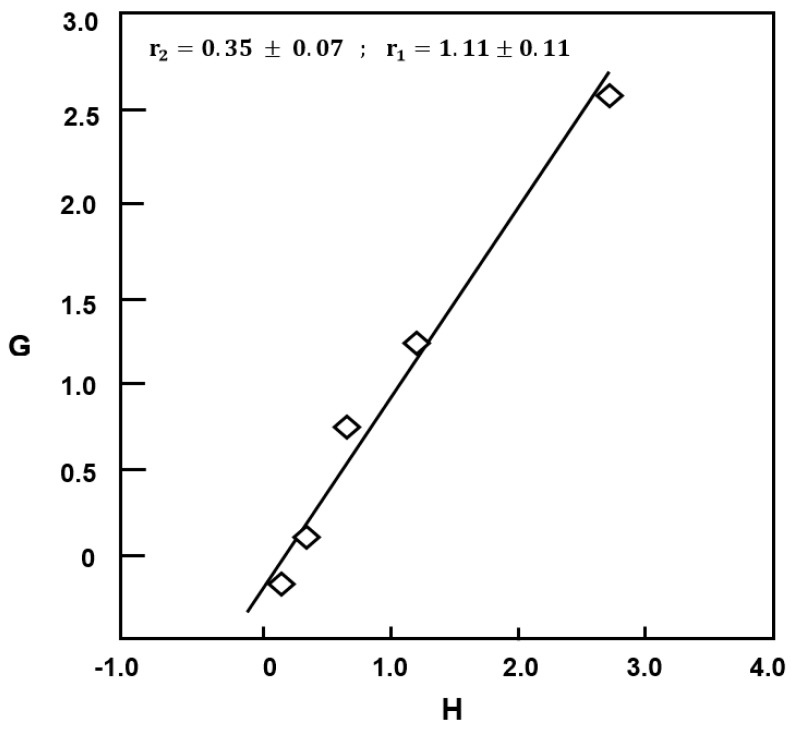
Fineman–Ross plot indicating variation of *G* vs. *H* for copolymerization of EUGMA with MMA.

**Figure 11:**
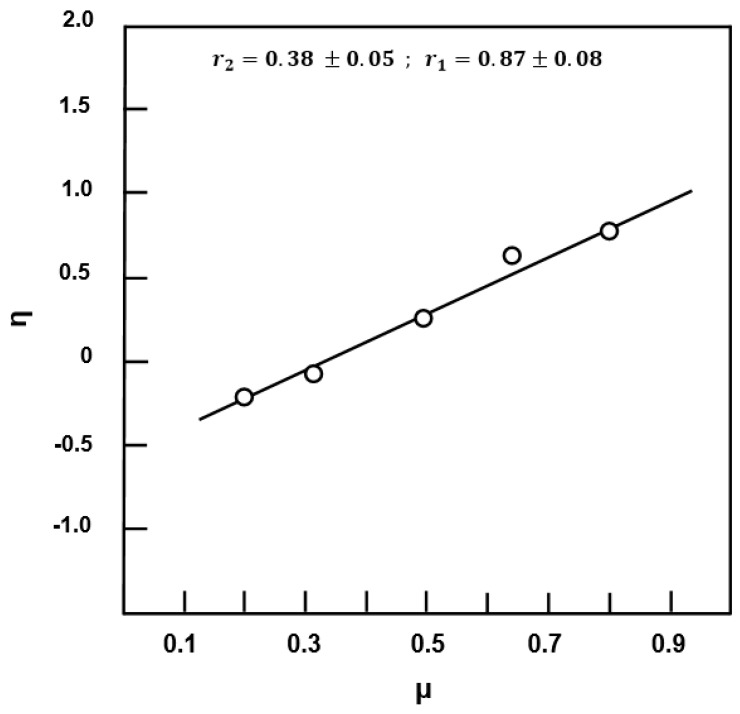
Kelen–Tüdős plot indicating variation of *η* vs. *μ* for copolymerization of EUGMA with MMA.

*r*_2_ and *r*_1_ can be estimated from the intercept and the slope of the linear curve of *G* vs. *H* of Figure 10.

(5) $$\eta =\left[r_{1}+\frac{r_{2}}{\alpha }\right]\mu -\frac{r_{2}}{\alpha }$$

where

(6) $$\eta =\frac{G}{\alpha +H};\;\mu =\frac{H}{\alpha +H}$$

with

(7) $$\alpha ={\left(H_{\min}H_{\max}\right)}^{1/2}$$

where *H*_min_ and *H*_max_ are the lowest and highest values of *H* from the Fineman–Ross equation. The data can be plotted in linear form as in Equation (5). Therefore, $r_{2}$ can be calculated from the intercept of the linear curve of *η* vs. *µ* of Figure 11. Then, $r_{1}$ can be obtained from the slope of the curve, as shown in the equations above. The averaged values of *r*_1_ and *r*_2_ obtained by the three methods are gathered for comparison in Table 4. As can be seen from these data the reactivity values obtained by the three methods are comparable, and show that the distribution of the comonomeric units in the polymer chains should be random, with a tendency toward alternation. Although the steric hindrance of the eugenyl substituent which hinders the incorporation of its self or the other monomer in the growing chain, the higher polarity of EUGMA which contains hydroxyl group carries it and promotes the easier incorporation of MMA. Comparable results were also obtained by Deshpande et al. [36] in the copolymerization of MMA with alkylmethacrylate monomer containing a hydroxyl group such as 2-hydroxypropyl methacrylate (HPMA) by a free radical polymerization route in which the reactivity values were 1.06 and 0.40 for HPMA and MMA, respectively.

#### 3.2.3. Distribution of the Dyad Monomer Sequences

**Figure 12:**
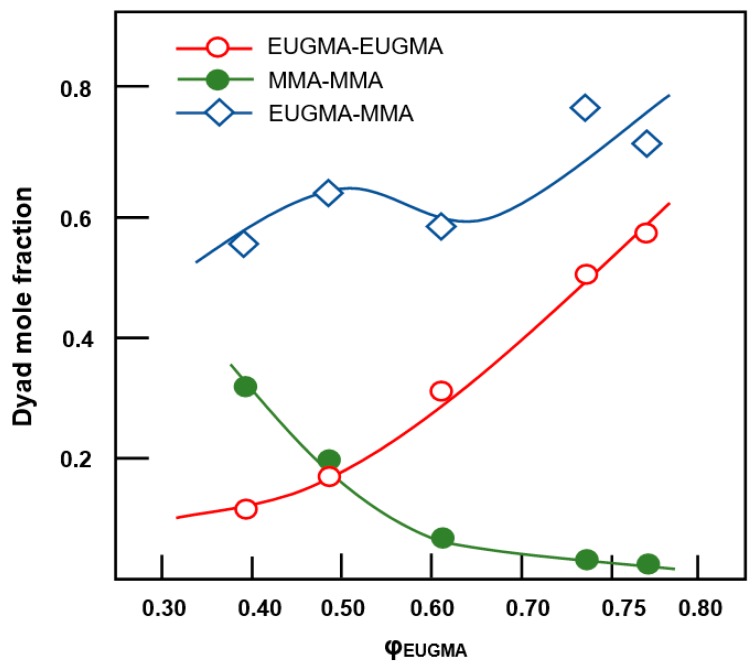
Distribution of dyad sequences in the copolymer vs. EUGMA mole fraction.

The distribution of the dyad monomer sequences EUGMA–EUGMA, MMA–MMA and EUGMA–MMA were calculated using the method proposed by Igarashi [37]:

(8) $$X=\varphi_{1}-\frac{2\varphi_{1}\varphi_{2}}{1+{\left[{\left(2\varphi_{1}-1\right)}^{2}+4r_{2}r_{1}\varphi_{1}\varphi_{2}\right]}^{1/2}}\;\;\;\;\;\;$$

(9) $$Y=\varphi_{2}-\frac{2\varphi_{1}\varphi_{2}}{1+{\left[{\left(2\varphi_{1}-1\right)}^{2}+4r_{2}r_{1}\varphi_{1}\varphi_{2}\right]}^{1/2}}$$

(10) $$Z=\frac{4\varphi_{1}\varphi_{2}}{1+{\left[{\left(2\varphi_{1}-1\right)}^{2}+4r_{2}r_{1}\varphi_{1}\varphi_{2}\right]}^{1/2}}$$

where *X*, *Y* and *Z* are the mole fractions of the EUGMA–EUGMA, MMA–MMA and EUGMA–MMA dyads in the copolymer, respectively and *φ*_1_ and *φ*_2_ are the EUGMA and MMA mole fraction in the copolymer, respectively. Using the data of Table 3 and Equations (8)–(10), the variation of these different dyad fractions are calculated and the results obtained are displayed in Figure 12. These results confirm the conclusion drawn by the reactivity ratio in which the distribution of the monomeric units in the polymer chains is random, with a tendency toward alternation. Indeed, a high content of EUGMA–MMA heterodyads compared with those of their corresponding homogeneous dyads EUGMA–EUGMA and MMA–MMA is revealed. These results also show that the mole fraction of EUGMA–EUGMA dyads and EUGMA–MMA increased with the EUGMA content in the copolymer.

### 3.3. Thermal Analysis

#### 3.3.1. DSC Analysis

**Figure 13:**
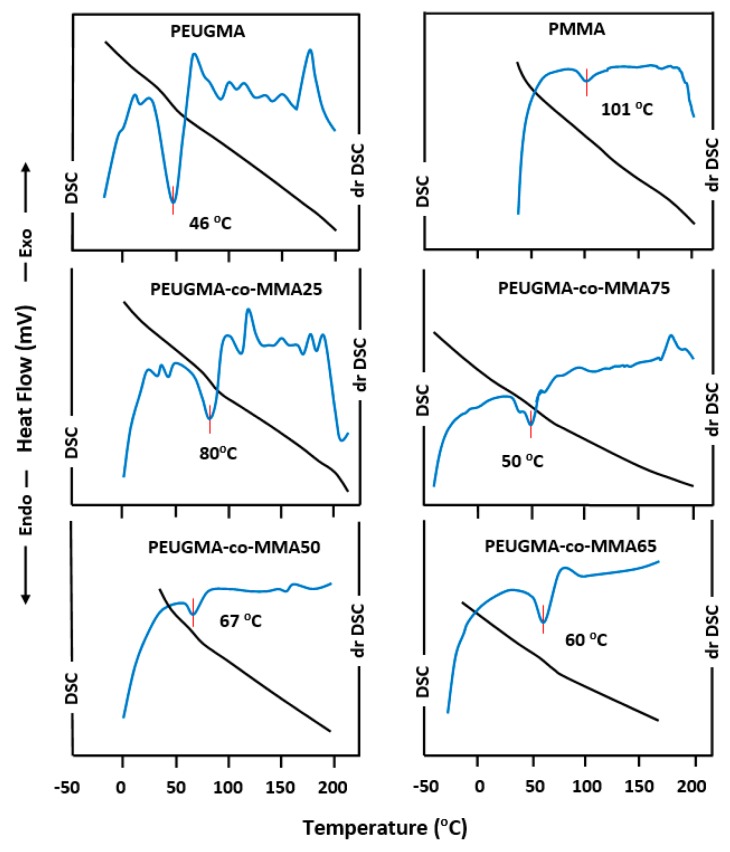
Differential scanning calorimetry (DSC) and derivative-DSC thermograms of PMMA, PEUGMA and PEUGMA-co-MMA with different EUGMA content.

A uniform thermal history across all specimens was ensured by presenting thermograms with traces of the second run after quenching from temperatures slightly above *T_g_*. As presented in Figure 13, the thermogram of the PMMA homopolymer shows that *T_g_* occurs at 101 °C, which is in agreement with the literature [38,39], while that of the PEUGMA homopolymer shows a *T_g_* at 46 °C. On the other hand, the thermal curves of PEUGMA-co-MMA copolymers show a dependence of the thermal properties on the EUGMA unit incorporated in the copolymer.

Table 5 summarizes the *T_g_* values deducted. As indicated by this table, the glass transition behavior of PMMA is significantly influenced by the EUGMA content, in which the *T_g_* value from PMMA to PEUGMA-co-MMA copolymers decreased from 101 to 46 °C when the bulky monomeric unit incorporated varied from 0 to 100 wt %. The Fox equation [40] was also used to predict the glass transition temperature of a copolymer. According to different authors [41,42,43,44], statistical or random copolymers are characterized by a good correlation between the experimental values of *T_g_* and those calculated from the Fox equation. On the other hand, the values of *T_g_* for alternative copolymers deviate from those calculated.

(11) $$\frac{1}{T_{g(Cop)}^{Cal}}=\frac{w_{1}}{T_{g(1)}}+\frac{w_{2}}{T_{g(2)}}$$

where $T_{g\left(\mathrm{cop}\right)}^{\mathrm{Cal}}$, *T_g_*_(1)_ and *T_g_*_(2)_ are the calculated glass transition temperatures of copolymer, homopolymer (1) and homopolymer (2), respectively. *w*_1_ and *w*_2_ are the weight fraction of comonomers (1) and (2).

Using this criterion and replacing the letters by the values in Equation (11), it was possible to calculate the $T_{g\left(cop\right)}^{Cal}$ value for each sample. The obtained results were added to this table for comparison. As can be seen from these data, the values of *T_g_* calculated, $T_{g\left(cop\right)}^{Cal}$ negatively deviated from those obtained experimentally, and the resulting difference decreased from 24.0 to 1.7 °C with an increase of EUGMA content in the copolymer.

**Figure 14:**
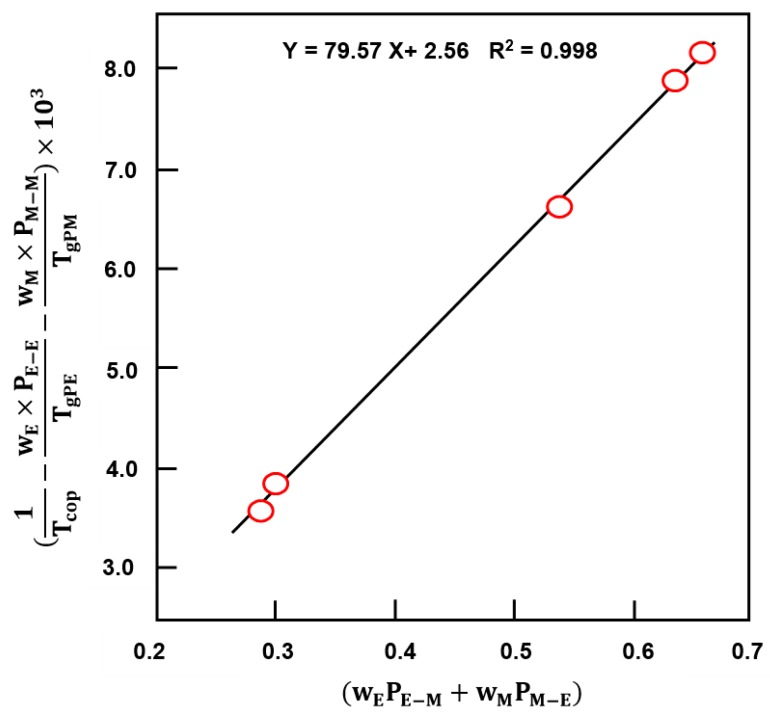
Linearized expression of Johnston’s treatment to the EUGMA–MMA copolymer system.

Taking into account the dominance of the different neighboring interactions, the behavior of glass transitions can be determined from the contribution of three comonomer pairs in terms of dyads sequencing [45], i.e., MMA–MMA, EUGMA–EUGMA and MMA–EUGMA or EUGMA–MMA. In this way, the effect of the microstructure of the copolymer on its glass transition has been considered by Johnston [46] and Barton. [47] Indeed, the Johnston approach is based on the free volume theory which is considered as an extension of that of Fox [40]. San Roman et al. [45] suggested that a linear expression of Johnston’s can be written as:

(12) $$\frac{1}{T_{g}cp}-\frac{w_{E}P_{E-E}}{T_{gPEUGMA}}-\frac{w_{M}P_{M-M}}{T_{gPMMA}}=\frac{w_{E}P_{E-M}+w_{M}P_{M-E}}{T_{g\left(E-alt-M\right)}}$$

where *T_g cp E−M_* is the glass transition temperature of the poly(EUGMA-*co*-MMA), *w_E_* and *w_M_* are the average weight fractions of EUGMA and MMA comonomer units in the copolymer chains, *T_gPEUGMA_* and *T_gPMMA_* are the glass transition temperatures of PEUGMA and PMMA homopolymers, respectively, and *T_g_*_(*E−alt−M*)_ that of the alternating copolymer; *P_E−E_*, *P_E−M_*, *P_M−E_* and *P_M−M_* refer to the probabilities of having various linkages defined statistically by Equations (13) and (14) [46]

(13) $$P_{E-M}=1-P_{M-M}=\frac{1}{\left(1+r_{2}X\right)}$$

(14) $$P_{M-E}=1-P_{E-E}=\frac{1}{\left(1+r_{1}X^{-1}\right)}$$

where *X* is the EUGMA/MMA concentration ratio in the feed. *P_E−E_*, *P_E−M_*, *P_M−E_* and *P_M−M_* symbolize the probabilities of EUGMA–EUGMA, EUGMA–MMA, MMA–EUGMA and MMA–MMA linkages, respectively. These parameters were calculated from Equations (13) and (14), and their application in the linearized Johnston’s treatment as shown in Figure 14, gives a straight line with a slope indicating a *T_g_*_(*E−alt−E−M*)_ of 79.6 °C. The *T_g_* values of the copolymer with different compositions calculated from this method are shown in Table 5 and reveal a best coherence with those obtained experimentally compared to those of the Fox approach.

#### 3.3.2. TGA Analysis

**Figure 15:**
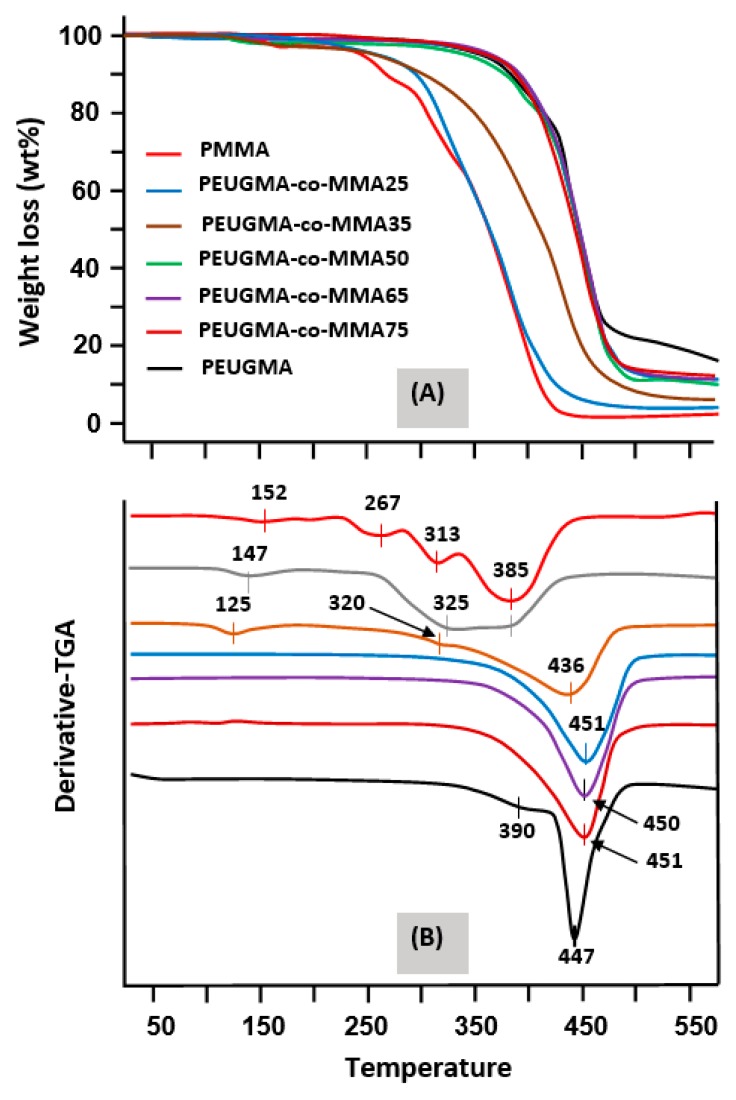
TGA (**A**) and derivative-TGA (**B**) thermograms of pure PMMA, pure PEUGMA and PEUGMA-co-MMA with different EUGMA content.

The effect of the EUGMA content incorporated in the copolymer on the thermal degradation of PMMA was examined by the TGA method, and the thermograms obtained in a nitrogen gas atmosphere are gathered for comparison in Figure 15A,B. As shown in the thermal curve of the PMMA, three main loss-of-weight regions were observed. Among the many investigations that have been devoted to the thermal degradation of PMMA, similar results were obtained by Kashiwagi et al. [48] using the same route of preparation and allowed for an acceptable interpretation of the observed phenomena.

According to these investigators, the first decomposition step, which is localized between 165 and 235 °C, would be initiated by scissions of head-to-head linkages (H–H). The bond dissociation energy of H–H linkages was estimated to be less than that of a C–C backbone bond owing to a large steric hindrance and inductive effect of vicinal ester groups. The second step between 283 and 340 °C was described as being initiated by scissions at unsaturated ends (resulting from termination by disproportionation) involving a homolytic scission β to the vinyl group. Finally, the last step that starts at around 330 °C would be initiated by random scission within the polymer chain.

According to the literature [49], the nature of the alkyl group in poly(alkylmethacrylate)s also determines the mode of the thermal decomposition of these types of polymers. Indeed, at heating temperatures above 200 °C, an unzipping reaction occurred in the almost practical and quantitative production of monomer [26] from the poly(alkylmethacrylate)s of higher ester groups. However, these tended to decompose only partially to a regenerating monomer. Poorer yields of monomer were generally obtained when passing up the homologous series and from primary to secondary and tertiary esters. Thus, poly(*n*-butylmethacrylate) produces about 50% of *n*-butylmethacrylate, while poly(*tert*-butylmethacrylate) yields about 1% of *tert*-butylmethacrylate and an almost quantitative amount of isobutene and poly(methacrylic anhydride) [26]. The decomposition of poly(*tert*-butylmethacrylate) involves ester decomposition to produce poly(methacrylic) acid, which then loses water to form the anhydride.

PEUGMA, in which the ester group is much more cluttered than that of *tert*-butyl, shows a thermogram with only one main decomposition step that starts at 340 °C, well beyond that of PMMA.

A comparison of the thermal decomposition of the two poly(methacrylic) esters reveals that the substitution of the methoxy group of PMMA with another bulker type such as eugenoxy considerably increases the thermal stability of the poly(methacrylic) ester, and the decomposition process is prolonged by about 100 °C. On the other hand, the TGA thermograms of the PEUGMA-co-MMA copolymers reveal an important shift in the decomposition temperature toward the right with regard to pure PMMA, varying between 260 °C for the copolymer containing 39.79 mol % of EUGMA (PEUGMA-co-MMA25) to 350 °C for that containing 77.05 mol % units of this bulky monomer (PEUGMA-co-MMA75). As can be seen from these data, the incorporation of EUGMA units in the PMMA leads to an increase in the thermal stability of PMMA. The jump in the increase of thermal stability of PMMA due to the addition of a small amount of EUGMA is considered an exploit.

The activation energy *E*_a_ of the pure PMMA, pure PEUGMA and PEUGMA-co-MMA copolymers was estimated from the pseudolinear portions of the thermal decomposition of each sample using the integral method proposed by Broido [50]:

(15) $$Ln[Ln(\frac{1}{Y})]=-\frac{E_{a}}{RT}+C\;with\;Y=\frac{W_{T}-W_{\infty }}{W_{0}-W_{\infty }}$$

where *Y* represents the fraction of the sample not yet decomposed, and *w_o_*, *w*_∞_, and *w_T_* are the initial weight, final weight and weight at a certain temperature, respectively.

**Figure 16:**
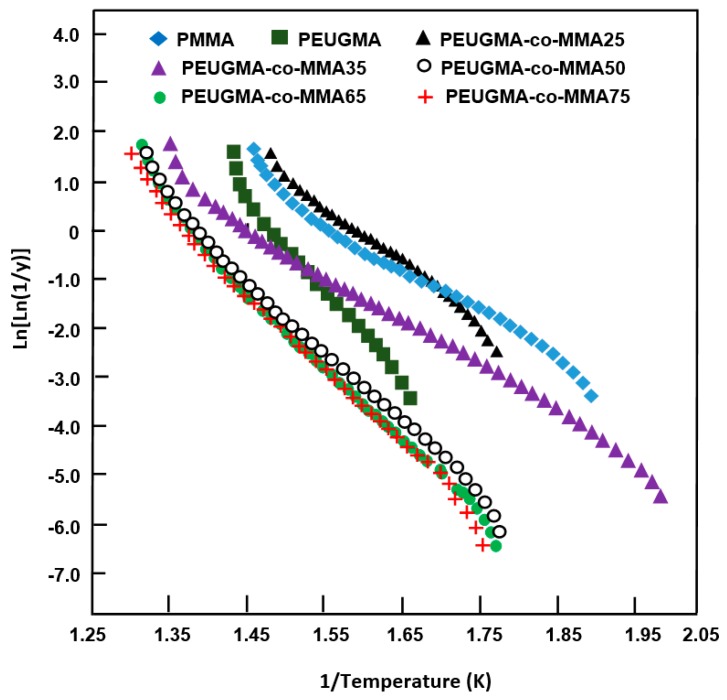
Variation of Ln[Ln(1/Y)] vs. the inverse of temperature plotted for PMMA, PEUGMA and their corresponding copolymers.

The variation of $Ln[Ln(\frac{1}{Y})]$ vs. the inverse of the temperature plotted for pure PMMA, pure PEUGMA, and their corresponding copolymers in Figure 16 is linear. Thus, *E*_a_ of the thermal decomposition process was deduced from the respective slopes. As can be seen from these curve profiles, the activation energy of pure PMMA was estimated as 82 kJ·mol^−1^, which is lower than that obtained by the Kissinger equation reported in the literature (113.0 kJ·mol^−1^) [51]. Next, 128 kJ·mol^−1^ [52] was obtained for PMMA by the free radical polymerization route, while that of pure PEUGMA was superior at 153.11 kJ·mol^−1^.

The activation energy increased from 109.60 to reach a maximum of 138.20 kJ·mol^−1^ when the EUGMA content in the copolymer changed from 39.79 to 77.05 mol %. This result indicates that the incorporation of an amount of EUGMA units in the PMMA main chain enhanced the thermal stability of this polymer. The increase in the *E*_a_ value when the EUGMA units incorporating the copolymers increased is probably due to the higher lower energy required for bond scission and the unzipping of PEUGMA-co-MMA copolymers.

#### 3.3.3. DART-ToF-MS Analysis

**Figure 17:**
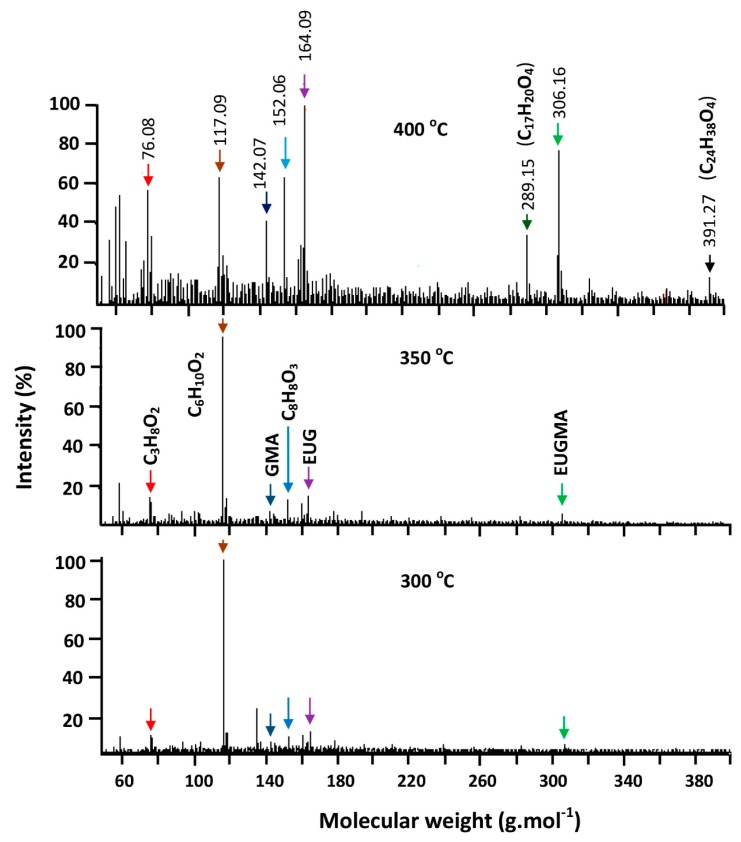
Thermograms of isothermal decomposition of PEUGMA indicating intensity of the absorption signal attributed to resulting products vs. molecular weight.

**Figure 18:**
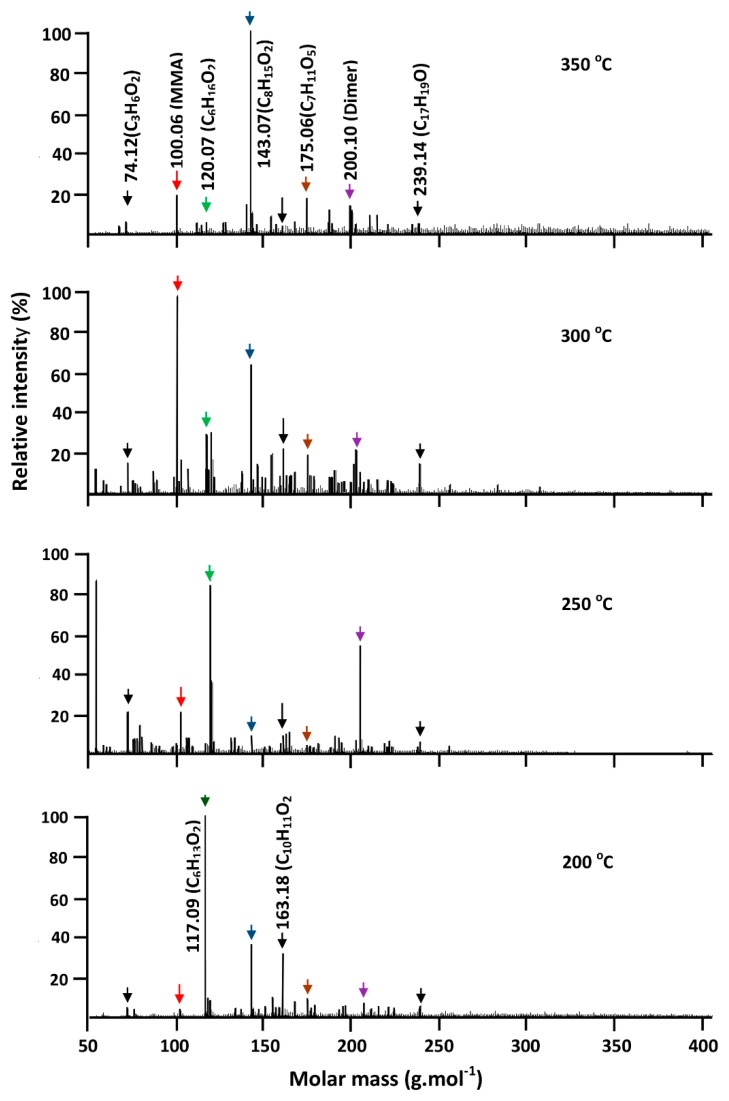
Thermograms of isothermal decomposition of PMMA indicating intensity of absorption signal attributed to resulting products vs. molecular weight.

**Figure 19:**
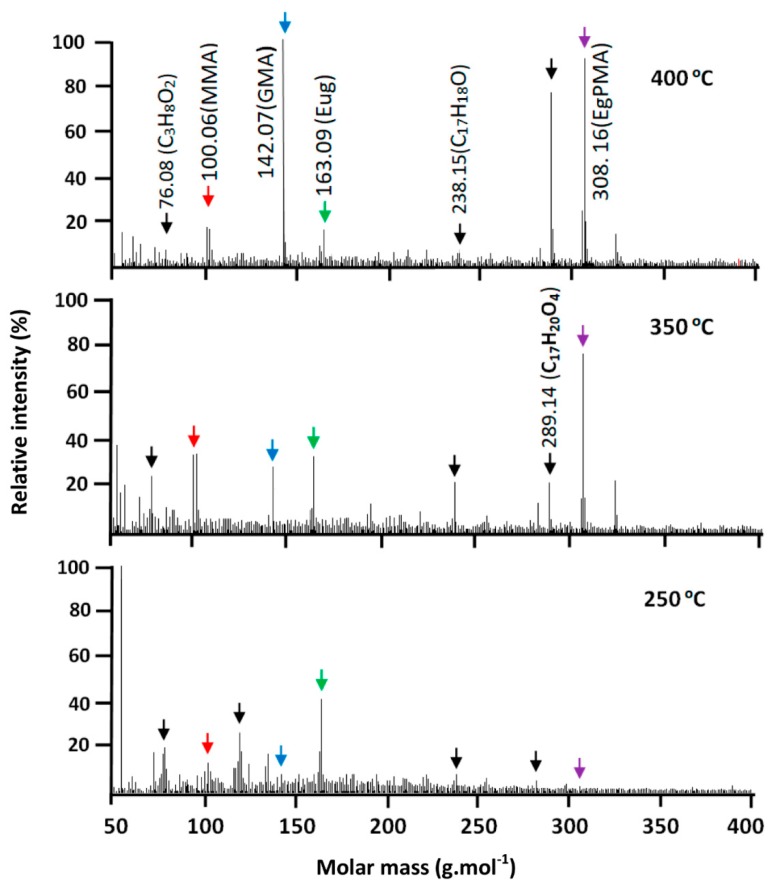
Thermograms of isothermal decomposition of PEUGMA-co-MMA indicating intensity of absorption signal attributed to resulting products vs. molecular weight.

The isothermal degradation of the synthesized materials in a helium atmosphere was investigated by the DART-ToF-MS method at 200, 250, 300, 350, 400 and 450 °C. This revealed the results of the decomposition, in which the more striking values are presented in Figure 17, Figure 18 and Figure 19. As can be seen from these traces in Figure 17, the decomposition of PEUGMA essentially produced the monomer and started materials such as GMA and EUG. These data also revealed that the regeneration of monomer began at 350 °C and reached a maximum at 450 °C, in which 23.53 mol % of EUGMA, 15.06 mol % of GMA and 15.06 mol % of EUG were regenerated. This result is similar to those obtained using TGA, in which the thermogram showed only an ultimate degradation temperature, indicating the production of an important ratio of EUGMA at this temperature.

The other resulting products which are the isomers of C_17_H_20_O_4_, C_8_H_9_O_3_, C_6_H_10_O_2_ and C_3_H_8_O_2_, are attributed to a probable fragmentation of the starting bulky monomers and also the polymer backbone, followed by rearrangement reactions that occurred during the degradation process. During this step, an important ratio of 1,2-propanol (76.08 g·mol^−1^) and its isomers was released.

PMMA that was submitted to the same conditions showed the thermograms of Figure 18. The data obtained at 200 °C revealed no signals indicating the regeneration of MMA, while three principal signals attributed to the isomers of C_6_H_12_O_2_ (116, 09 g·mol^−1^), C_7_H_10_O_3_ (142.07 g·mol^−1^) and 163.18 (C_10_H_11_O_2_) g·mol^−1^ are present in this thermogram. On the other hand, the thermogram obtained at 300 °C shows an intense signal at 100.06 g·mol^−1^ with an unsaturation degree of 1.5, indicating the chemical structure of MMA, and thus confirming the results previously obtained by TGA. Other less intense signals were observed at this temperature, revealing the formation of isomers of which the more striking are C_7_H_10_O_3_ (142.07 g·mol^−1^), C_9_H_11_ (119.07 g·mol^−1^), C_8_H_10_O_3_ (154.12 g·mol^−1^), C_7_H_10_O_5_ (174.06 g·mol^−1^), dimer (200.10 g·mol^−1^), C_9_H_15_O_4_ (190.09 g·mol^−1^) and C_17_H_18_O (238.15 g·mol^−1^) resulting from the fragmentation of the polymer chain, followed by a rearrangement reaction.

Concerning the PEUGMA-co-MMA, Figure 19 shows a thermogram of the thermal decomposition of this copolymer containing 13.40 mol % of MMA, taken as an example from the series of synthesized copolymers. As can be seen from these traces, practically no signal of the starting monomer EUGMA is observed at temperatures less than 250 °C. At this temperature, only the EUG is mostly regenerated and small amounts of MMA and GMA are also observed. In addition, other signals attributed to an eventual fragmentation of the bulky substituent are observed in this thermogram. This led principally to the formation of isomers of C_3_H_8_O_2_ (76.08 g·mol^−1^) and C_9_H_11_ (119.17 g·mol^−1^).

Conversely, an intense signal is observed at 308.16 g·mol^−1^ under a temperature of 300 °C. This indicates that the regeneration of a significant ratio of EUGMA as well as achieving a relatively small signal at 100.06 g·mol^−1^ are the results observed due to different polymer chain scissions; the aforementioned small signal is usually assigned to the regeneration of MMA and other products. The thermogram obtained at 450 °C shows the maximum EUGMA amount regenerated and a small amount of MMA; this reflects the initial composition of this copolymer (13.40 mol % of MMA). As for the homopolymers and copolymers, the degradation process also led to the production of molecules such as the isomers of C_3_H_8_O_2_, C_17_H_18_O and C_17_H_20_O_4_.

## 4. Conclusions

We conclude this study by presenting the significant results that were achieved. First, the successful synthesis of EUGMA via the etherification of a eugenol phenolic hydroxyl group with a GMA epoxy-ring opening route. Second, the polymer and copolymers that resulted from the polymerization and copolymerization of EUGMA and MMA showed molar masses that were relatively high and decreased as the starting EUGMA increased in the reaction mixture. The molar mass distribution varied randomly with the comonomers. The variation of the different dyad fractions in the copolymer calculated from the Igarashi method confirm the conclusion drawn by the reactivity ratio in which the distribution of the monomer units in the copolymer chains is random, with a tendency toward alternation.

The thermal properties of these materials investigated by the DSC method revealed for the PEUGMA a *T_g_* of 46 °C, which increased as the number of MMA units increased in the copolymer. The *T_g_* values of the copolymer with different compositions calculated from the Johnston approach reveal a best coherence with those obtained experimentally compared to those of the Fox approach.

The thermal decomposition of polymers and copolymers studied by TGA showed for PEUGMA only one main decomposition step, which started at 340 °C, well beyond that of PMMA. On the other hand, PEUGMA-co-MMA copolymers revealed an important shift in the decomposition temperature with regard to pure PMMA, which varied between 260 °C and 350 °C according to the composition.

The activation energy of homopolymers and copolymers which had been estimated from the Broido relationship revealed for PMMA a value of 81.55 kJ·mol^−1^, which is largely inferior to that of PEUGMA (153.20 kJ·mol^−1^). An important thermal stability of PMMA was observed with the incorporation of EUGMA and increased with the EUGMA units in the copolymer. A DART-ToF-MS analysis revealed that the isothermal decomposition of PEUGMA led to a regeneration of the monomer and raw materials such as GMA and EUG. During this regeneration, the maximum amount of these components was achieved at 450 °C. Conversely, under the same conditions, PMMA regenerated maximum MMA at 300 °C. With regard to the copolymers, only small amounts of MMA and GMA were regenerated at 250 °C, and the maximum number of isomers of C_3_H_8_O_2_ was obtained.

## Figures and Tables

**Table 1:** Preparation conditions of PEUGMA, PMMA and PEUGMA-co-MMA.

Polymer and Copolymer	Monomer (g)		Monomer (Mole Fraction)	
	EUGMA	MMA	EUGMA	MMA
PEUGMA	3.06	-	1.00	-
PEUGMA-co-MMA25	0.51	0.49	0.25	0.75
PEUGMA-co-MMA35	0.62	0.38	0.35	0.65
PEUGMA-co-MMA50	0.75	0.25	0.50	0.50
PEUGMA-co-MMA65	0.85	0.15	0.65	0.35
PEUGMA-co-MMA75	0.90	0.10	0.75	0.25
PMMA	-	3.00	-	1.00

**Table 2:** Yields, average molar mass and polydispersity index of the PEUGMA, PMMA and PEUGMA-co-MMA copolymers.

Copolymer	Yield (wt %)	${\bar{M}}_{n}$ (g·mol^−1^) × 10^5^	${\bar{M}}_{w}$ (g·mol^−1^) × 10^5^	I
PEUGMA	-	1.08	3.21	2.96
PEUGMA-co-MMA25	10.0	1.22	2.99	2.45
PEUGMA-co-MMA35	10.6	1.28	2.46	1.92
PEUGMA-co-MMA50	11.4	1.32	2.81	2.12
PEUGMA-co-MMA65	11.2	1.36	2.94	2.16
PEUGMA-co-MMA75	12.0	1.48	3.32	2.25
PMMA	-	1.78	4.40	2.47

**Table 3:** Comparative results of the copolymer compositions of PEUGMA-co-MMA determined by ^1^HNMR and ultraviolet (UV)-visible methods.

Copolymer	Composition (mol %)					
	^1^H NMR		UV-Visible		Average	
	EUGMA	MMA	EUGMA	MMA	EUGMA	MMA
PEUGMA-co-MMA25	38.52	64.42	41.06	58.94	39.79	60.21
PEUGMA-co-MMA35	47.22	52.78	50.18	49.82	48.70	51.30
PEUGMA-co-MMA50	58.80	44.20	64.82	35.18	61.81	38.19
PEUGMA-co-MMA65	73.18	26.82	74.82	25.18	74.00	26.00
PEUGMA-co-MMA75	78.18	12.82	75.92	24.08	77.05	22.95

**Table 4:** Reactivity ratios of the copolymerization of EUGMA with MMA.

Reactivity	Mayo–Lewis	Fineman–Ross	Kelen–Tüdős	Average
*r*_1_	1.05 ± 0.07	1.11 ± 0.11	0.87 ± 0.08	1.01 ± 0.09
*r*_2_	0.32 ± 0.10	0.35 ± 0.07	0.38 ± 0.05	0.35 ± 0.07

**Table 5:** Experimental and calculated glass transition temperatures of PMMA, PEUGMA and PEUGMA-co-MMA.

Polymer and Copolymer	W_E_ *	W_M_ *	$T_{g}^{Exp}$ (°C)	$T_{g}^{Fox}$ (°C)	$\Delta T_{g}$ (°C)	$T_{g}^{Johnston}$ (°C)	$\Delta T_{g}$ (°C)
PMMA	0	1.0	101	101	-		
PEUGMA-co-MMA25	0.67	0.33	80	56.0	24.0	74.0	6
PEUGMA-co-MMA35	0.75	0.25	73	53.2	19.8	68.0	5
PEUGMA-co-MMA50	0.83	0.17	67	51.0	16.0	61.0	6
PEUGMA-co-MMA65	0.90	0.10	60	48.6	16.4	55.0	5
PEUGMA-co-MMA75	0.91	0.09	50	48.3	1.7	53.0	3
PEUGMA	1.0	0	46	46	-	-	-

* W_E_ and W_M_ are the average weight fractions of EUGMA and MMA comonomer units in the copolymer chains.
